# The effects of socioeconomic deprivation on long‐term outcomes in Parkinson’s disease and frontotemporal lobar degeneration syndromes

**DOI:** 10.1002/alz.092378

**Published:** 2025-01-09

**Authors:** Anna Jane Dreyer, Alexander G Murley, Ian Coyle‐Gilchrist, Katherine Stockton, Negin Holland, Marta Camacho, Matthew A Rouse, Michelle Naessens, Carol Brayne, Roger A Barker, Caroline H. Williams‐Gray, James B Rowe

**Affiliations:** ^1^ University of Cambridge, Cambridge United Kingdom; ^2^ Cambridge University Hospitals NHS Foundation Trust, Cambridge United Kingdom; ^3^ Norfolk and Norwich University Hospital, Norwich United Kingdom; ^4^ Cambridge Public Health, University of Cambridge, Cambridge United Kingdom; ^5^ MRC Cognition and Brain Sciences Unit, University of Cambridge, Cambridge United Kingdom

## Abstract

**Background:**

Socioeconomic deprivation has been associated with shorter survival and an earlier loss of functional independence in patients with Alzheimer’s disease and dementia. However, the effect of deprivation on outcomes in Parkinson’s disease (PD) and syndromes associated with frontotemporal lobar degeneration (FTLD) are largely unknown. Prognosis in these neurodegenerative diseases is variable and difficult to predict despite multiple known sociodemographic, clinical and genetic predictors. Using data from three long‐term prospective incident cohorts in the UK, we conducted a survival analysis to investigate the association of the deprivation in one’s residential area with outcomes after symptom onset and diagnosis.

**Method:**

Within known geographical settings patients with FTLD‐associated syndromes (bvFTD, PPA, PSP, CBS; *N* = 283) were recruited in three waves (2013‐2014, 2017‐2018, 2021‐2022) as part of PIPPIN. Similarly, patients with PD were recruited as part of CamPaIGN (*N* = 140) from 2000‐2002, and PICNICS (*N* = 280) from 2008‐2013. The Index of Multiple Deprivation (IMD) centile was assigned to each participant according to their residence at the time of diagnosis and dates of care home admission and death recorded from NHS records. Using Cox proportional hazards regression, we investigated the univariable and multivariable associations between IMD and (1) absolute survival (time to death) (2) independent survival (time to care home admission/death) (3) remaining cognitively healthy in PD (time to dementia diagnosis).

**Result:**

IMD did not predict absolute or independent survival, in any of the three cohorts, in univariable or multivariable analyses. In univariable models, IMD predicted time to PD dementia diagnosis in PICNICS, but not in the other cohorts. Multivariable models showed that poorer outcomes were associated with sociodemographic (older age, being male), clinical (disease severity, cognitive deficits, depression, FTLD diagnosis) and genetic variables (APOE, GBA), but not IMD.

**Conclusion:**

To live in an area of higher socioeconomic deprivation is not associated with poorer long‐term survival in patients with FTLD‐associated syndromes or PD. In patients with PD, living in an area of high deprivation predicted shorter time to dementia diagnosis. These reassuring results might reflect universal healthcare coverage (i.e., NHS), with specialist services for patients able to mitigate deprivation effects.

